# Elevated expression of circulating miR876-5p is a specific response to severe EV71 infections

**DOI:** 10.1038/srep24149

**Published:** 2016-04-07

**Authors:** Robert Y. L. Wang, Kuo-Feng Weng, Yhu-Chering Huang, Chih-Jung Chen

**Affiliations:** 1 Department of Biomedical Sciences, College of Medicine, Chang Gung University, Taoyuan, 33302, Taiwan; 2 Research Center for Emerging Viral Infections, College of Medicine, Chang Gung University, Taoyuan, Taiwan; 3 Division of Pediatric Infectious Diseases, Department of Pediatrics, Chang Gung Memorial and Children’s Hospital, Linkuo, Taiwan

## Abstract

Human enterovirus 71 (EV71) is a major causative agent of hand, foot, and, mouth disease, accounting for more than 65% of recent outbreaks. Following enteroviral infection, the host responses are crucial indicators for the development of a diagnosis regarding the clinical severity of EV71 infections. In this study, we implemented NanoString nCounter technology to characterize the responses of serum microRNA (miRNA) profiles to various EV71 infection diseases. Upon EV71 infection, 44 miRNAs were observed in patients with EV71 infections, with at least a 2-fold elevation and 133 miRNAs with a 2-fold reduction compared with the same miRNAs in healthy controls. Further detailed work with miR876-5p, a 9.5-fold change of upregulated miR-876-5p expression was observed in cases with severe EV71 symptoms, revealed that *in vitro* and *in vivo* knockdown of miR876-5p reduced viral RNA in cultured cells, and attenuated the severity of symptoms in EV71-infected mice. Altogether, we demonstrated that the elevated expression of circulating miR876-5p is a specific response to severe EV71 infections.

Human enterovirus 71 (EV71) is a single-strand positive-sense RNA virus that belongs to the Enterovirus genus of the Picornaviridae family^1^,^2^. EV71 is a highly contagious disease that mainly affects children younger than 5 years, and is among the frequently observed pathogens of hand, foot, and mouth disease (HFMD)^2^,^3^. Common transmission routes are fecal–oral and respiratory. Primary EV71 infection causes viral replication in the mucosa tissue surrounding the respiratory or gastrointestinal tract^4^. Clinical manifestations can vary from subclinical, uncomplicated mucocutaneous diseases and aseptic meningitis to severe diseases including rhombencephalitis, neurogenic pulmonary edema, and cardiopulmonary dysfunctions, and even death^5^. Since the late 1990s, large epidemic outbreaks of EV71 associated with severe neurological complications and high mortality rates have occurred in Taiwan, Singapore, Japan, and other areas in the Asia-Pacific^5^,^6^,^7^,^8^,^9^. In 1998, EV71 caused a large-scale epidemic in Taiwan, with 405 documented severe cases and 78 deaths^1^,^10^,^11^.

Akin to other RNA viruses that cause diseases in humans, EV71 relies on host genetic factors to invoke different physical responses to the same virulence factors of the pathogen. For example, tumor necrosis factor promoter type II, HLA-33A, and HLA-DR17 have all been associated with EV71 susceptibility^6^. Another example is the association of the endothelial nitric oxide synthase G894T gene polymorphism with EV71 infection, and its possibility of being a susceptibility factor in the development of EV71 infection^12^. In addition, host factors are crucial for determining the EV71 cell tropism. Several neurotropic EV71 strains, derived from a clinical encephalitis sample, replicated efficiently in the neuroblastoma (SF268) and rhabdomyosarcoma (RD) cell lines, indicating that EV71 can replicate in a wide range of cells. Cellular receptors and internal ribosomal entry site transacting factors were 2 major host factors that determined the EV71 tropism^13^,^14^,^15^.

MicroRNAs (miRNAs) are groups of small, single-stranded, non-coding RNAs. Their major identified function involves mediating the posttranscriptional silencing of target genes. Overall, miRNAs regulate cell processes by inactivating specific cellular mRNAs by binding to complementary sites in the 3′ untranslated region (UTR) of target genes, thereby inhibiting protein expression and inducing mRNA degradation of the target gene^16^. Numerous studies have demonstrated that miRNAs are key regulators of diverse biological processes, including cancer, development, and even immune responses^17^,^18^. In addition, many special miRNAs have been reported to participate in the cross-talk regulation between a host and the pathogen caused by viral infections, and to play critical roles in the host response to viral infections^19^. Circulating miRNAs are packaged into exosome-like particles that are detectable in serum. The circulating miRNAs are resistant to RNase digestion, and are highly stable in serum and various body fluids^20^. This unique characteristic of circulating miRNAs makes them ideal and powerful biomarkers for the detection of infectious diseases. For example, altered serum miRNA expression profiles were reported in patients infected with EV71 and coxsackievirus A16^21^. Cui *et al*. found that the detection of a group of miRNAs could facilitate distinguishing patients with viral infections from healthy controls.

The pathogenesis of an EV71 infection is complex, with the central nervous system (CNS) likely being the main target. The clinical features of an EV71-caused severe disease are identifiable by neurological complications, such as brainstem encephalitis, aseptic meningitis, and myelitis^22^,^23^. Neurologic pulmonary edema is thought to be the main pathogenic cause in fatal cases of EV71 infection. It has been reported that robust virus replication within infected host cells, in combination with tissue damage and the induction of toxic inflammatory cytokines and cellular immunity, is a possible process of pathogenesis. The reports mentioned have indicated that the EV71 virus initially proliferates in a specific tissue or organ before viremia, and further damage of target organs upon viral infection occurs. In response to viral infection, the host may alter several specific biological pathways, such as apoptosis, differentiation, development, metabolism, and control of homeostasis. Recent evidence has indicated that miRNAs can regulate multiple functionally related genes involved in a specific signaling pathway. The regulation of particular biological pathways is the major result of miRNA–mRNA interactions; therefore, the extensive deregulation of miRNA expression may reflect various cellular defensive responses through inflammation as well as bacterial and viral infections.

In this study, we identified 177 miRNAs associated with different EV71 infection stages in children, in which no differentiated miRNA expression was identified in patients with mild and severe EV71 infections, indicating a correlation between circulating miRNAs and the disease spectrum of EV71 infections. The knockdown of miR876-5p resulted in a reduction of EV71 viral RNA in neuroblastoma (SF268) cells. We also demonstrated that the inhibition of miR876-5p can attenuate the severe symptoms of EV71 infection in mice. Overall, we concluded that miR876-5p is secreted in response to EV71 infection.

## Results

### EV71 infection cohort and features of patients undergoing miRNA profiling study

During the study period, 40 children with HFMD or herpangina were enrolled in this cohort, of whom 19 (47.5%) had EV71 that was isolated from throat and/or rectal swab cultures. For the miRNA study, random samples of 8 cases were selected from participants with laboratory-confirmed EV71 infections. Four patients were diagnosed with severe infections, and the other 4 patients were diagnosed with mild infections. All of the participants were previously healthy children aged 1.5–9.2 years (median, 2.7 y). The duration of symptoms before admission to hospital ranged from 2 to 5 days (median, 3 d). One patient died from cardiopulmonary failure. Four patients with mild infection recovered after treatment. The healthy controls were recruited randomly from children who visited clinics for a regular health checkup without exhibiting the clinical symptoms of any infectious disease The clinical features of 8 EV71-infected patients with distinct severities and 4 healthy control children undergoing serum global microRNAs array can be found as Supplementary Table S1.

### Profiling the expression pattern of serum miRNA from children with mild and severe EV71 infection

**Figure 1:**
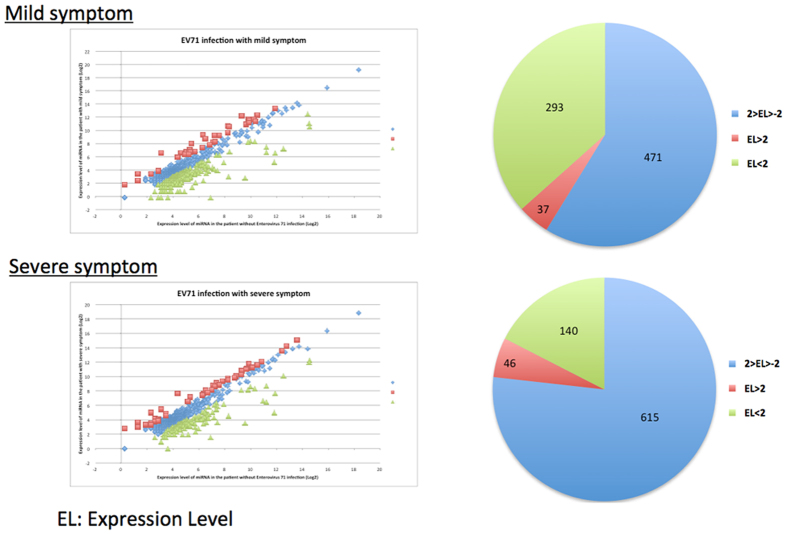
NanoString profiling indicates differential serum expression levels of circulating miRNAs in healthy controls and children with mild or severe symptoms of EV71. The miRNA profile results for the serum RNA of healthy patients versus patients with mild EV71 symptoms (**A**) and healthy patients versus patients with severe EV71 symptoms (**B**) were log2-transformed and quantile-normalized. Values at least one standard deviation above the background were included. “EL” indicates the expression level.

RNA was isolated from patients with mild and severe EV71 infections and healthy controls by using the consistent purification technique, described in the Materials and Methods section, in addition to simultaneous isolation to avoid batch effects. To identify the candidate circulating miRNAs with levels that were significantly altered in response to EV71 infection, the nanoString hybridization platform was used. As reported in the literature, the nanoString platform involves performing microscopy to count fluorescently bar-coded probes, detecting miRNA molecules without the amplification or introduction of position-dependent effects. This system can simultaneously detect the quantitative expression levels of 800 miRNA molecules^24^. We first screened the altered serum miRNAs levels in response to EV71 infections in mild or severe cases and compared them with the healthy controls. The data were processed using several normalization strategies, including quantile normalization and normalization to certain invariant miRNAs. We required the differential expression of miRNAs among mild or severe EV71 infections and healthy controls to exhibit more than a 2-fold difference for a specific miRNA. Of the 800 miRNAs incorporated in this assay, 471 miRNAs did not reveal a differential expression between mild EV71 infections and healthy controls, and 615 miRNAs did not indicate a differential expression between the severe EV71 infections and healthy controls. In patients with mild EV71 infections, the levels of 37 miRNAs were observed to be significantly elevated, and those of 293 miRNAs to be reduced in relation to the levels detected in the healthy controls (Fig. 1A). In a similar manner, the levels of 46 miRNAs were observed to have increased significantly, and those of 140 miRNAs decreased from patients with severe EV71 infections in relation to the levels detected in the healthy controls (Fig. 1B).

### Differential expression of microRNAs in mild and severe EV71 infections compared with healthy controls

We then queried the data set to identify individual miRNA species that were differentially regulated specifically in response to mild and severe EV71 symptoms. To fulfill this objective, we screened for serum miRNA tightly associated with EV71 infections, for which the differential expression of miRNAs had more than a 2-fold difference between the EV71 infections and healthy controls. We observed 117 human miRNAs to have a significantly differentiated expression in response to a mild EV71 infection. Among them, the levels of 14 miRNAs were observed to have at least a 2-fold elevation, and those of 103 miRNAs to be reduced compared with the levels of the healthy controls (see Supplementary Table S2). In a similar manner, 30 miRNAs had an upregulated expression, and another 30 miRNAs had a downregulated expression in severe EV71 infection compared with the expression in healthy controls (see Supplementary Table S3). All of these differentially expressed miRNAs were detected at least 2 standard deviations above the background (by using the NanoString array system), in descending order, and were believed to be closely associated with the severity of EV71 infections. None of the differentiated expressed miRNAs were identified in the patients with either mild or severe EV71 infections, indicating a correlation between circulating miRNAs and the disease spectrum of EV71 infections.

### Validation of differentially expressed miRNAs by individual stem-loop quantitative PCR

**Figure 2:**
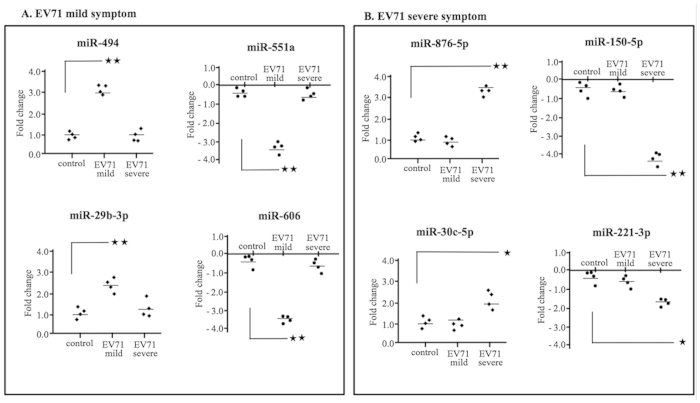
The qRT-PCR validation of selected microRNAs of patients with mild EV71 symptoms (n = 4), severe EV71 symptoms (n = 4), and healthy controls (n = 4). The relative expression in mild (**A**) and severe (**B**) EV71 symptoms and healthy controls was calculated using the comparative Ct (2-∆ ∆Ct) method. The miRNA expression level was normalized against U6 miRNA. PCRs were run for duplicates for each subject. The graphs represent one-way ANOVA and Dunnett’s multiple-comparison tests of significantly up- or downregulated miRNAs in patients with mild or severe EV71 symptoms. Each data point indicates an individual subject. Horizontal bars denote the mean of miRNA expression in each group. *P < 0.01; **P < 0.001.

To further validate the NanoString microRNA profiling results, we chose a group of 8 miRNAs comprising has-miR-494, has-miR-29b-3p, has-miR-551a, has-miR-606, has-miR-876-5p, has-miR-30c-5p, has-miR-221-3p, and has-miR-150-5p for follow-up analysis by using an individual quantitative PCR assay. We also considered has-miR-494 and has-miR-876-5p, which have an obvious fold change (an 8.2-fold change of upregulated miR494 expression was observed in cases with mild EV71 symptoms, and a 9.5-fold change of upregulated miR-876-5p expression was observed in cases with severe EV71 symptoms) in response to EV71 infections, and have not been reported in other studies. We then used a scatter plot to represent the relative expression levels of these 8 miRNAs (Fig. 2). The quantification of the 8 small RNAs, along with snRNA U6, was performed in patients and healthy controls. The expression of has-miR-494, has-miR-29b-3p, has-miR-876-5p, and has-miR-30c-5p was more abundant in the blood serum of patients with mild or severe EV71 infections, whereas has-miR-551a, has-miR-606, has-miR-221-3p, and has-miR-150-5p were less abundant in the blood serum of patients with mild or severe EV71 infections than they were in the blood serum of healthy controls (Fig. 2). These results revealed a consistent correlation between the quantities of miRNA transcripts, as measured using both NanoString and qRT-PCR assays.

### MiR-876-5p and miR-150-5p were detected in media from EV71-infected neuroblastoma cells

**Figure 3:**
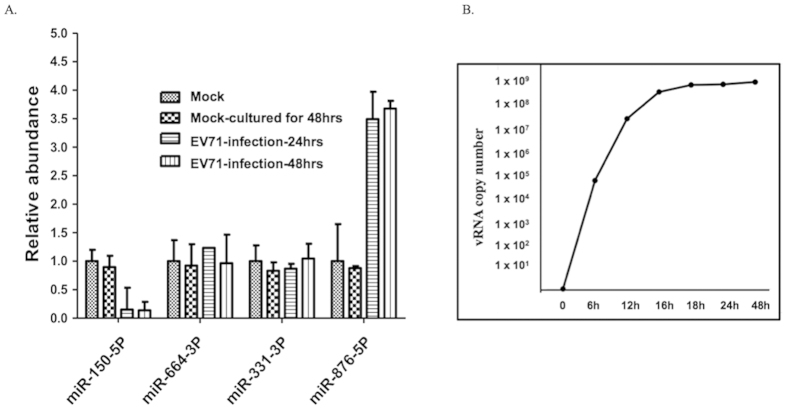
The miRNA 876-5p expression was upregulated during EV71 infection. (**A**) Human neuroblastoma SF268 cells were mock- or EV71-infected at an MOI of 1 for 24 h and 48 h, followed by the collection of the cultured medium for the analysis of 4 miRNAs by using specific primers, as indicated. The expression of miR-150-5p, miR-664-3p, miR-331-3p, and miR-876-5p was evaluated through qPCR. The data represent the means ± SEM from 3 independent experiments for *in vitro* study. (**B**) The growth kinetics of EV71 in SF268 cells.

**Figure 4:**
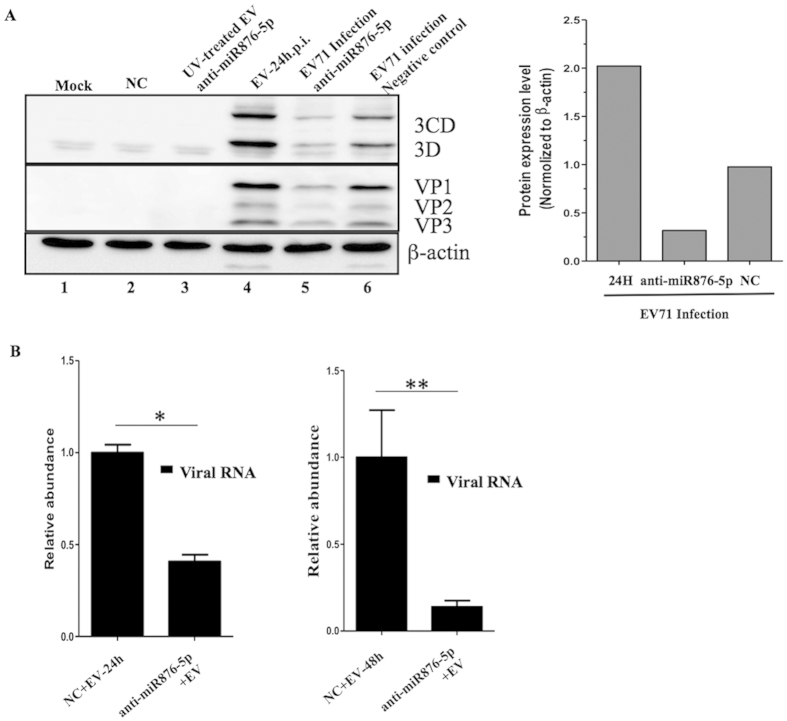
Knockdown of miR-876-5p attenuated EV71 viral replication. (**A**) The SF268 cells were transfected with anti-miR-876-5p (hairpin inhibitor) for 24 h, followed by infection with EV71 for 24 h. At the end of the period, the cell lysates were harvested for the investigation of viral proteins, 3CD/3D and VPs, using specific antibodies, as indicated. The right-hand panel represents the quantitative protein expression level normalized to β-actin. (**B**) Effects of anti-miR-876-5p inhibitor on the expression of viral RNA. The SF268 cells were transfected with anti-miR-876-5p (hairpin inhibitor) for 24 h, followed by infection with EV71 for 24 h or 48 h. The cells were harvested for the isolation of total RNA. Viral RNA expression was evaluated by RT-qPCR. The data represent 3 independent experiments, and ^*^P < 0.05 and ^**^P < 0.01 compared with miRNA hairpin inhibitor negative control (NC).

To examine the potential of identified miRNAs as EV71-induced clinical progress biomarkers, we first validated the expression levels of extracellular miRNAs in the cell culture system. To this end, we collected the cultured media from mock- and EV71-infected neuroblastoma SF268 cells. We quantitated the expression levels of 4 miRNAs in the cultured media by using specific primers: The expression of miR-876-5p increased in EV71-infected neuroblastoma cells compared with the cells of uninfected controls, whereas miR-150-5p expression decreased in the cultured medium of EV71-infected cells. By contrast, no differentiated expression levels were observed in other miRNAs, such as miR-664-3p and miR-331-3p, in EV71-infected cells (Fig. 3A), which is consistent with the NanoString miRNA profiling results. Also, the measurement of virus growth curve was shown to provide the evidence that this cultured cells were indeed infected with EV71 (Fig. 3B). Second, we evaluated the functional role of miR-876-5p when it was involved in the EV71 infection cycle *in vitro*. Treatment using an anti-miR-876-5p inhibitor resulted in a significant reduction of viral proteins 3C and 3D as well as VPs (Fig. 4A). In a similar manner, a significant decrease in the expression of EV71 viral RNA comparable to viral protein findings (Fig. 4B) was observed. These data strongly indicated that miR-876-5p expression is upregulated after EV71 infection. Therefore, it is likely that the secretion of miR-876-5p is a specific response to EV71 infection *in vivo*.

### Anti-miR-876-5p treatment reduces EV71-related symptoms in infected mice

**Figure 5:**
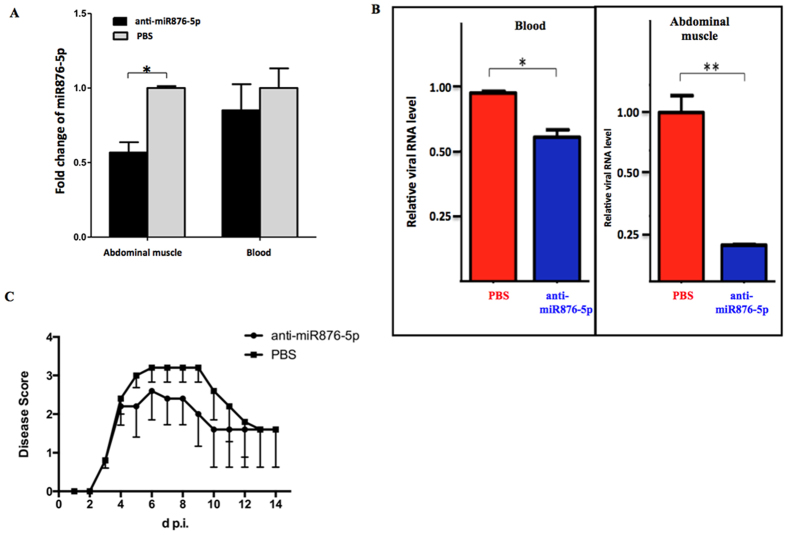
The effect of anti-miR876-5p inhibitor treatment on viral RNA abundance and virus-induced diseases in EV71-infected mice. (**A**) Mice were treated with the miRNA hairpin inhibitor negative control in PBS or anti-miR876-5p inhibitor for 24 h, followed by RNA isolated from the site of injection in the abdominal muscle or collected blood. The expression level of miR-876-5p was evaluated by Q-RT-PCR. The data represent 3 independent experiments, and *P < 0.05 compared with miRNA hairpin inhibitor negative control. (**B**) Abdominal muscle and blood samples were collected from anti-miR876-5p or PBS-negative control treated mice at 24 h after EV71 infection. The viral RNA levels were determined using Q-RT-PCR and calculated as relative abundances. Error bars represent SD (n = 5, ^*^P < 0.05, ^**^P < 0.01, *t* test). (**C**) The EV71-induced diseases on anti-miR876-5p inhibitor or PBS-negative control treated mice were monitored daily and scored (0 = health, 1 = limb weakness, 2 = single hind limb paralysis, 3 = double hind limb paralysis, 4 = death) from 1 to 14 d postinfection. The error bars represent SEM (n = 5).

The potential anti-EV71 efficacy of anti-miR-876-5p treatment was first tested in EV71-infected mice. The mice were intraperitoneally injected with anti-miR876-5p (miR876-5p hairpin inhibitor) or PBS for 24 h prior to EV71 infection. The EV71 we used was a mouse-adapted strain (EV71 mp4), which has been reported to induce neurological diseases and death on the virus-infected ICR mice^24^. Our results revealed that anti-miR876-5p significantly reduced the miR-876-5p levels in the abdominal muscles and slightly reduced the levels in blood (without significantly) of the treated mice at 24 h post-injection, compared with the levels of PBS-treated mice (Fig. 5A). As displayed in Fig. 5B, the significantly reduced viral RNA abundance of anti-miR876-5p-treated mice compared with the PBS treatment mice were observed within the abdominal tissue samples at 5 days postinoculation (dpi), indicating that anti-miR-876-5p reduced the viral loads at the site of EV71 infection. In addition, the anti-miR-876-5p-treated mice were found to exhibit fewer EV71-induced neurological symptoms compared with the PBS-treated mice from Day 5 to Day 10 after EV71 infection (Fig. 5C), as indicated using averaged disease scores (0 = healthy; 1 = hind limb weakness; 2 = single hind limb paralysis; 3 = double hind limb paralysis; 4 = death). Therefore, we concluded that anti-miR-876-5p treatment reduced EV71 pathogenicity in virus-infected mice.

## Discussion

To the best of our knowledge, this is the first comprehensive miRNA profiling of differentially expressed serum miRNAs in the serum of patients with mild and severe EV71 infections compared with the serum of healthy controls, especially in children. Moreover, we validated the differentiated expression pattern of miR-876-5p in EV71-infected neuroblastoma cells and mice. Circulating miRNAs have recently been considered potentially diagnostic and prognostic biomarkers, because human fluids, including plasma/serum, urine, cerebrospinal fluid, saliva, and even tears are easily retrievable from people^25^. In addition, it has been documented adequately that miRNAs detected in the serum/plasma are highly stable under several harsh environments, including extreme pH levels, high heat and boiling, and multiple freeze-thaw cycles^26^. Because of these unique characteristics, circulating miRNAs are believed to be powerful biomarkers for the early diagnosis of different diseases such as cancer (lung cancer, breast cancer, and colon cancer)^27^,^28^,^29^ and certain microbial infections^28^,^30^. A combination of miR-17, miR-20a, miR-106a, and miR-376c indicated a high level of accuracy for detecting H7N9 viral infection^30^, and an altered expression pattern of miR-361-5p, miR-899, and miR-576-3p was reported to distinguish patients with pulmonary tuberculosis from healthy people^28^. In this study, we identified a focused group of 8 miRNAs comprising has-miR-494, has-miR-29b-3p, has-miR-551a, has-miR-606, has-miR-876-5p, has-miR-30c-5p, has-miR-221-3p, and has-miR-150-5p, which demonstrated an expression pattern in patients infected with EV71 as distinct from the controls. Instead of predicting the potential diagnosing ability of using the combination of several miRNAs identified from the serum, we focused on the obvious upregulated expression of miRNA and miR-876-5p, exploring the putative miRNA functional responses to EV71 infections.

The targeted genes from identified miRNAs were classified according to their function in the context of a larger network of proteins that interact to accomplish at least one biological process at the cellular level. The PANTHER classification system was employed for this purpose. The data suggested that the identified miRNAs were major regulators or receivers in 2 biological processes, the metabolic process and cellular process, indicating that alterations in cellular homeostasis are the main response to viral infections. In addition, functional enrichment analysis on these cellular genes revealed various overrepresented pathways, including the coagulation cascade pathway (miR-494, see Supplementary Fig. S1), Mucin type O-glycan biosynthesis pathway (miR-551a, see Supplementary Fig. S2), PI3K-Akt pathway (miR-876-5p, see Supplementary Fig. S3), and MAPK signaling pathway (miR-150-5p, see Supplementary Fig. S4), which are critical cellular pathways related to EV71 infection. We summarized the interactions among these enriched Kyoto Encyclopedia of Genes and Genomes pathways to construct several protein–protein interaction networks by using the DAVID functional annotation tool^31^. Several miR-494 targeted genes were involved in the complement and coagulation cascades pathways, including the tissue factor pathway inhibitor (TFPI), SERPIND1, and PROS1 (see Supplementary Fig. S1). The significantly elevated expression of miR-494 in response to a mild EV71 infection resulted in the downregulation of TFP1, SERPIND1, and PROS1 proteins, and led to altered inflammation, prostaglandin biosynthesis, and nitric oxide biosynthesis outcomes. By contrast, the most significantly reduced expression of miR-551a in response to mild EV71 infections regulated several genes, which are related to Type II diabetes, mellitus, or mucin type O-glycan biosynthesis pathways (see Supplementary Fig. S2). The glucosamyl (N-acetyl) transferase 1 (GNCT1), a member of the beta-1,6-N-acetylglucosaminyltransferase gene family, is the primary target gene of miR-551, as shown in this pathway. Moreover, the significantly elevated expression of miR-876-5p in response to a severe EV71 infection resulted in the downregulation of the cyclic-AMP responsive element-binding protein (CREB5) protein, which has been shown to affect the PI3K-Akt signaling pathway, including CREB5 and IL6R proteins (see Supplementary Fig. S3). In a similar manner, the most significantly reduced expression of miR-150-5p in response to a severe EV71 infection occurred in genes related to the MAPK signaling pathway, including CACN, FGFR, TGFBR, HGK, MKP, p38, NLK, cPLA2, and p53 proteins (see Supplementary Fig. S4). These results suggested that the dysfunction of miR-876-5p and miR-150-5p induced cell apoptosis in response to severe EV71 infections.

We also observed a significantly reduced expression of miR-150-5p in the serum of a patient with a severe EV71 infection. According to our results, many genes involved in the p38 MAPK signaling pathway are regulated by miR-150-5p, indicating that the p38 MAPK pathway is closely associated with severe EV71 infections. Furthermore, p38 MAPK is not only activated through viral infection but also has crucial functions that are involved in the host immune response and in the regulation of cell survival after a viral infections^32^,^33^. The activation of p38 MAPK signaling enables cells to respond to external signals, and triggers numerous biological effects leading to the maintenance of cellular homeostasis. Hence, the MAPK signaling pathway-related genes, including CACN, FGFR, TGFBR, HGK, MKP, p38, NLK, cPLA2, and p53 proteins, were regulated by miR-150-5p, indicating that the response and regulation of the expression of key genes for survival in patients with severe EV71 infection is highly complex. Our results implied that the combination of differentiated expressions of miRNAs (upregulation of miR-876-5p and downregulation of miR-150-5p) should provide an indication of circulating miRNA patterns detected in the serum of patients with mild and severe EV71infections.

In conclusion, our data suggested that altered miRNA expression levels detected in the serum from patients with an EV71 infection can regulate a number of key genes involved in the major signaling pathways associated with the diagnosis of EV71 infection. In this study, we have known that, in EV71 severe infection, the altered expression of miR-876-5p (upregulation) and miR-150-5p (downregulation) regulated genes that are related to PI3K-Akt and MAPK signaling pathways. Both of the signaling pathways regulate the cell cycle and cell apoptosis indicating viral infection caused the altered in cellular mircroRNAs regulation and subsequently affected the host genes expression levels resulting in the cell apoptosis. Finally, we focused on miR-876-5p, which can lead to distinguishing the symptoms of severe EV71 infections. Our findings showed that an elevated expression of miR876-5p is a specific response to a severe EV71 infection.

## Methods

All experimental protocols were approved by the Research Ethics Board of Chang Gung Memorial Hospital.

### Study population

The study was conducted in a university-affiliated teaching hospital (Chang Gung Memorial Hospital) in Northern Taiwan during July 2011 and September 2012. The hospital is a 3700-bed facility, which provides primary to tertiary care to children. Child blood samples were drawn from the antecubital veins of patients with EV71 infection or healthy volunteers in accordance with the guidelines approved by the institutional review board of Chang Gung Memorial Hospital in 2011. During the study period, children aged 12 years or younger hospitalized in Chang Gung Memorial Hospital with a presumptive diagnosis of enteroviral infections (i.e., HFMD and herpangina) were enrolled in the study if inform consent was obtained from their parents or guardians. The medical information of each enrolled participant was collected using a chart review of information including demographic data, clinical manifestations, laboratory values, treatments, and outcomes. The informed consent was obtained from all subjects. Serum samples were retrieved at enrollment, and throat and rectal swabs were obtained for the identification of EV71. The swab sample was part of routine medical practice. Swab specimens were inoculated into human embryonic fibroblast (MRC-5), Madine-Darby canine kidney, HEp-2, and RD cells. All cultures were observed daily for cytopathic effects (CPEs). Indirect fluorescent staining with panenteroviral antibody (Chemicon International, Temecula, CA, USA) was performed to identify the enterovirus when CPEs involved more than 50% of the cell monolayer. The identification of EV71 was confirmed through neutralization with EV71-specific immune sera (Chemicon International, Temecula, CA, USA).

### Enteroviral disease symptom definitions

Participants with laboratory-confirmed EV71 infections were further categorized into 3 groups of mild infection, aseptic meningitis, and severe infection. A mild EV71 infection was defined as a febrile illness with mucocutaneous lesions such as herpangina and HFMD. Patients in this group displayed no neurological symptoms or hemodynamic changes. The herpangina symptoms were well-characterized vesicular enanthem and ulcers of the fauces and soft palate, accompanied by a fever, sore throat, and decreased appetite. The HFMD symptoms were oral ulcers that occurred primarily on the buccal mucosa and tongue, accompanied with typical vesicular rashes that were most commonly on the extensor surfaces of the hands, feet, knees, and buttocks. Patients with aseptic meningitis experienced irritability and headaches as well as cerebrospinal fluid pleocytosis (>5 × 10^6^ leukocytes per liter), but no altered level of consciousness or focal signs. Severe EV71 infection included encephalitis, polio-like syndrome, encephalomyelitis, pulmonary complications, and heart failure. Patients with encephalitis had an altered level of consciousness in combination with cerebrospinal fluid pleocytosis. Patients with polio-like syndrome had acute limb weakness and decreased reflex and muscle strength. Moreover, patients with encephalomyelitis had both encephalitis and polio-like syndrome. Pulmonary complications included pulmonary edema, hemorrhaging, or diffuse ground–glass infiltrations observed on roentgenographies with decreased PaO_2_/FiO_2_ < 300. Acute heart failure was characterized based on observations of decreased contractility on echocardiography, arrhythmia, an enlarged heart, and elevations in cardiac enzymes, which are markers of cardiac damage.

### Sample collection and miRNA extraction

The global miRNAs profiles of the collected blood samples were analyzed using the NanoString nCounter kit. Immediately after collection, plasma was isolated from the blood without anticoagulants through centrifugation. The supernatant was then left to clot overnight at room temperature. The clotted material was removed the next day through centrifugation under sterile conditions. Upon receipt at our laboratory, the serum was aliquoted into 1-mL units and stored at −80 °C. The collected sera were subjected to total RNA extraction by using the mirVana PARIS kit. The RNA was eluted in 150 μL of the Ambion elution buffer. The quality and quantity of isolated RNA were measured in a NanoVue spectrophotomer (GE Healthcare, Piscataway, NJ, USA). The RNA was extracted from 100 μL of individual serum samples, which were used for real-time reverse transcription polymerase chain reaction (RT-PCR) assay.

### MiRNA profiling using a NanoString array system

The NanoString nCounter Human miRNA Expression Assay Kit (http://www.nanostring.com) was used to profile 800 human and human-associated viral miRNAs. In total, 80–100 ng of RNA was used for the nCounter miRNA sample preparation reactions. All sample preparations were performed in accordance with the manufacturer instructions (NanoString Technologies). The prepared small RNA samples were ligated with a specific DNA tag onto the 3′ end of each mature miRNA. These tags were designed to normalize the Tms of the miRNAs and provide a unique identification for each miRNA species in the sample. After hybridization and the removal of excess capture and reporter probes, the purified ternary complexes were bound to the imaging surface before they were elongated and immobilized. The surface was then imaged using an nCounter digital analyzer. To account for slight differences in hybridization and purification, the data were normalized to average counts for all control spikes in each sample.

### NanoString data analysis: Normalization of expressing miRNA counts and selection of distinctive miRNAs

The expressing counts of miRNA in the 3 clinical samples acquired from Nanostring analysis were normalized with a geometric mean of the 100 miRNA with the highest counts by using nSolver Analysis (version 1.1) software. The normalized value of each miRNA was then converted into a Log2 value. The distinguished miRNAs expressed among the 3 clinical samples were determined through a comparison of the Log2 value of each miRNA. The Log2 value of the clinical sample from a patient mildly or severely infected with EV71 with more than a 2-fold or less than a 0.5-fold difference than the Log2 value from the clinical sample from a patient not infected with EV71s was grouped into EV71-regulated miRNAs. The miRNAs specifically expressed in the clinical sample from a patient mildly infected with EV71 were selected if they had an approximate 2-fold expression level difference with the miRNA expression level of the clinical sample from the patient not infected with EV71. However, the miRNA expression level difference of the clinical sample from the patient severely infected with EV71 was between 0.5- and 2-fold with that in the clinical sample from an uninfected patient. This approach was also used to select the distinctive miRNAs expressed in the clinical sample from patients infected with EV71 with severe symptoms.

### Individual RT-qPCR assays

We employed TaqMan miRNA assays (Applied Biosystems) to measure the differential expression of miRNAs identified using the NanoString nCounter system. The reverse-transcribed or reverse-transcribed and preamplified samples were assayed in accordance with the manufacturer protocol. Fifteen miRNAs were analyzed for the validation of nCounter results, and they were miRs -494, -551a, -29b-3p, -606, -876-5p, -150-5p, -30c-5p, -221-3p, and snRNA U6. Delta-delta Ct (Ct) analysis was performed, with a normalization to the geometric mean of the 15 least varying RNAs across samples and a comparison of each sample with the mean expression of the control samples. The results were independent of this normalization method, as established by an investigation of alternative normalization methods, including normalization to snRNA U6 (data not shown).

### Pathway categorization of predictive miRNA-targeting genes

Predictive miRNA-targeting genes were acquired from the TargetScanHuman (http://www.targetscan.org) Web site. The total content score of predictive miRNA-targeting genes less than −0.2 was selected for pathway categorization. Pathway categorization was archived using the Database for Annotation, Visualization and Integrated Discovery (DAVID) functional annotation tool and Protein ANalysis THrough Evolutionary Relationships (PANTHER) categorization system. The selected predictive miRNA-targeting genes were inputted into the DAVID gene list. The gene list was then annotated and viewed using a functional annotation chart. In addition to DAVID, the selected predictive miRNA-targeting genes were categorized and annotated into different families and subfamilies according to the molecular function, biological process, and pathway.

### Virus strain, virus infection, and cell line

The SF268 cells were cultured in Dulbecco’s Modified Eagle Medium (Gibco-BRL, Carlsbad, CA, USA) supplemented with 10% FBS (Gibco-BRL, Carlsbad, CA, USA), 100 units of penicillin, 50 μg/mL of streptomycin (Gibco-BRL, Carlsbad, CA, USA), and 24 mM of sodium bicarbonate (Sigma, St. Louis, MO, USA), and maintained at 37 °C in an atmosphere of 5% CO_2_. EV71 (Taiwan strain 2231) was amplified in RD cells, and the titers were measured using a plaque assay. For viral infection, the cells were washed with PBS and incubated in a serum-free medium 1 h prior to infection. Cells were infected through incubation with EV71 for 1 h at 37 °C with shaking every 15 min. Infected cells were washed with PBS and cultured in the complete medium, as described.

### Mice experiments

For *in vivo* mice experiments, the animal care and experimental procedures adhered to the protocol approved by the Institutional Animal Care and Use Committee of Chang Gung University (IACUC approval no.: CGU-14-113). Specific-pathogen-free 7-day-old ICR mice (n = 5 for each experimental group) were intraperitoneally injected with 50 μL of PBS-dissolved miRNA hairpin inhibitor Negative control (NC, purchased from Dharmacon, Lafayette, CO, USA) or PBS-dissolved anti-miR876-5p hairpin inhibitor (Dharmacon, Lafayette, CO, USA) at a dose of 25 mg/Kg by using a 30 G insulin syringe. At 24 h after anti-miR876-5p injection, the mice were intraperitoneally inoculated with 10^6^ PFU of EV71 mp4 virus (with a total volume of 50 μL) with a 30G insulin syringe. To detect RNA levels in the EV71-infected mice, blood and tissue samples were collected at 1 day postinfection after axilla dissection, and stored in a −70 °C freezer until the follow-up assays. To study the effect of anti-miR876-5p treatment on virus-induced diseases, the infected mice were monitored daily for clinical signs and disease scoring (0 = healthy, 1 = limb weakness, 2 = single hind limb paralysis, 3 = double hind limb paralysis, 4 = death) for 14 days after EV71 infection.

### Statistical analyses

All data are presented as the mean ± standard deviation. All variables were tested for normal distribution by conducting the Kolmogorov–Smirnov test. The Student *t* test was performed to compare the means of continuous variables and normally distributed data; otherwise, the Mann–Whitney *U* test was performed. The differences in the categorical variances were analyzed using Pearson’s chi-square test or Fisher’s exact test. Results with *P* < 0.05 were considered significant.

## Additional Information

**How to cite this article**: Wang, R. Y. L. *et al*. Elevated expression of circulating miR876-5p is a specific response to severe EV71 infections. *Sci. Rep.* **6**, 24149; doi: 10.1038/srep24149 (2016).

## Supplementary Material

Supplementary Information
