# Supplementary material for: Elevated expression of circulating miR876-5p is a specific response to severe EV71 infections
Source: Sci Rep. 2016 Apr 7;6:24149. doi: 10.1038/srep24149 (PMC4823700; doi:10.1038/srep24149)

Supplementary information:

Title:

Elevated expression of circulating miR876-5p is a specific response to severe EV71 infections

Robert YL Wang, Kuo-Feng Weng, Yhu-Chering Huang, Chih-Jung Chen

Table S1 - Table S3.

Fig. S1 - Fig.S4

Table S1. Clinical features of 8 EV71-infected patients with distinct severities and 4 healthy control children undergoing serum global microRNAs array.

Features	EV71 infected cases								Control patient
	Severe infection				Mild infection				
	Case I	Case II	Case III	Case IV	Case V	Case VI	Case VII	Case VIII	
Gender	Male	Female	Male	Male	Male	Male	Male	Female	
Age (years)	3.3	1.6	2.0	3.9	2.6	2.8	1.5	9.2	2.3
Underlying condition	Previous healthy	Previous healthy	Previous healthy	Previous healthy	Previous healthy	Previous healthy	Previous healthy	Previous healthy	healthy
Duration of symptom before hospitalization (days)	3	5	3	2	2	3	3	3	0
Clinical diagnosis	HFMD with encephalitis	HFMD with brain stem encephalitis and cardio-pulmonary failure	HFMD with brain stem encephalitis and cardio-pulmonary failure	HFMD with encephalitis	HFMD	Herpangina	HFMD	HFMD	none
Outcome	Survival without sequel	Survival with respiratory failure on mechanical ventilation, and limb paralysis	Died	Survival without sequel	Survival without sequel	Survival without sequel	Survival without sequel	Survival without sequel	none

Abbreviation: EV71, enterovirus 71; HFMD, hand-foot-and-mouth disease; VUR, vesicoureter reflux

Table S2. The list of up- and down-regulated expression of miRNAs detected from the blood serum of EV71-infected patient with mild symptom in comparison with healthy controls.

Gene Name	Accession Number	Target Sequence (5' to 3')	Mild[2],*	Control[1],*	Severe[3],*	Mild v.s. Control*	Severe vs. Control*
**Up-Regulated[4]**							
hsa-miR-494	MIMAT0002816	UGAAACAUACACGGGAAACCUC	9.33	6.30	6.25	3.03	-0.05
hsa-miR-29b-3p	MIMAT0000100	UAGCACCAUUUGAAAUCAGUGUU	8.00	5.40	5.87	2.60	0.47
hsa-miR-30e-5p	MIMAT0000692	UGUAAACAUCCUUGACUGGAAG	10.57	8.34	9.11	2.24	0.78
hsa-miR-19a-3p	MIMAT0000073	UGUGCAAAUCUAUGCAAAACUGA	9.25	7.21	7.73	2.04	0.52
hsa-miR-27b-3p	MIMAT0000419	UUCACAGUGGCUAAGUUCUGC	6.57	4.60	5.21	1.97	0.61
hsa-miR-19b-3p	MIMAT0000074	UGUGCAAAUCCAUGCAAAACUGA	11.66	9.83	10.49	1.82	0.65
hsa-miR-590-5p	MIMAT0003258	GAGCUUAUUCAUAAAAGUGCAG	7.00	5.28	5.29	1.73	0.01
hsa-miR-32-5p	MIMAT0000090	UAUUGCACAUUACUAAGUUGCA	6.72	5.03	5.05	1.69	0.02
hsa-miR-21-5p	MIMAT0000076	UAGCUUAUCAGACUGAUGUUGA	11.49	9.91	10.22	1.59	0.32
hsa-miR-302b-3p	MIMAT0000715	UAAGUGCUUCCAUGUUUUAGUAG	9.71	8.22	8.66	1.50	0.44
hsa-miR-199a-5p	MIMAT0000231	CCCAGUGUUCAGACUACCUGUUC	6.39	4.92	4.42	1.47	-0.50
hsa-miR-26b-5p	MIMAT0000083	UUCAAGUAAUUCAGGAUAGGU	13.36	11.89	12.30	1.47	0.41
hsa-miR-142-3p	MIMAT0000434	UGUAGUGUUUCCUACUUUAUGGA	11.69	10.25	10.48	1.43	0.23
hsa-miR-146a-5p	MIMAT0000449	UGAGAACUGAAUUCCAUGGGUU	7.86	6.84	6.17	1.02	-0.67
**Down-Regulated[5]**							
hsa-miR-137	MIMAT0000429	UUAUUGCUUAAGAAUACGCGUAG	2.97	3.98	4.36	-1.00	0.38
hsa-miR-374c-5p	MIMAT0018443	AUAAUACAACCUGCUAAGUGCU	2.39	3.44	3.55	-1.06	0.10
hsa-miR-564	MIMAT0003228	AGGCACGGUGUCAGCAGGC	1.80	2.86	3.66	-1.06	0.80
hsa-miR-1298	MIMAT0005800	UUCAUUCGGCUGUCCAGAUGUA	1.80	2.86	3.29	-1.06	0.43

hsa-miR-1272	MIMAT0005925	GAUGAUGAUGGCAGCAAAUUCUGAAA	1.80	2.86	2.96	-1.06	0.10
hsa-miR-2114-5p	MIMAT0011156	UAGUCCCUUCCUUGAAGCGGUC	2.97	4.08	4.36	-1.11	0.27
hsa-miR-613	MIMAT0003281	AGGAAUGUUCCUUCUUUGCC	2.61	3.73	3.77	-1.13	0.04
hsa-miR-1469	MIMAT0007347	CUCGGCGCGGGGCGCGGGCUCC	2.12	3.28	3.55	-1.15	0.27
hsa-miR-1203	MIMAT0005866	CCCGGAGCCAGGAUGCAGCUC	2.12	3.28	3.29	-1.15	0.01
hsa-miR-599	MIMAT0003267	GUUGUGUCAGUUUAUCAAAC	2.12	3.28	3.29	-1.15	0.01
hsa-miR-553	MIMAT0003216	AAAACGGUGAGAUUUUGUUUU	2.12	3.28	3.13	-1.15	-0.14
hsa-miR-3187-3p	MIMAT0015069	UUGGCCAUGGGGCUGCGCGG	2.80	3.98	4.13	-1.17	0.16
hsa-miR-924	MIMAT0004974	AGAGUCUUGUGAUGUCUUGC	2.39	3.60	3.96	-1.21	0.37
hsa-miR-1179	MIMAT0005824	AAGCAUUCUUUCAUUGGUUGG	2.39	3.60	3.87	-1.21	0.27
hsa-miR-193a-3p	MIMAT0000459	AACUGGCCUACAAAGUCCCAGU	2.39	3.60	3.66	-1.21	0.07
hsa-miR-1	MIMAT0000416	UGGAAUGUAAAGAAGUAUGUAU	2.39	3.60	3.55	-1.21	-0.05
hsa-miR-1267	MIMAT0005921	CCUGUUGAAGUGUAAUCCCCA	2.39	3.60	3.55	-1.21	-0.05
hsa-miR-634	MIMAT0003304	AACCAGCACCCCAACUUUGGAC	3.39	4.60	5.01	-1.21	0.41
hsa-miR-577	MIMAT0003242	UAGAUAAAAUAUUGGUACCUG	3.12	4.36	4.13	-1.24	-0.23
hsa-miR-1180	MIMAT0005825	UUUCCGGCUCGCGUGGGUGUGU	5.47	6.72	6.89	-1.25	0.18
hsa-miR-412	MIMAT0002170	ACUUCACCUGGUCCACUAGCCGU	2.61	3.86	4.05	-1.25	0.19
hsa-miR-491-5p	MIMAT0002807	AGUGGGGAACCCUUCCAUGAGG	2.61	3.86	3.96	-1.25	0.10
hsa-miR-297	MIMAT0004450	AUGUAUGUGUGCAUGUGCAUG	2.80	4.08	3.87	-1.28	-0.21
hsa-miR-3180	MIMAT0018178	UGGGGCGGAGCUUCCGGAG	1.80	3.08	2.96	-1.28	-0.12
hsa-miR-638	MIMAT0003308	AGGGAUCGCGGGCGGGUGGCGGCCU	3.71	5.03	5.13	-1.32	0.10
hsa-miR-512-5p	MIMAT0002822	CACUCAGCCUUGAGGGCACUUUC	2.12	3.44	3.42	-1.32	-0.02
hsa-miR-4741	MIMAT0019871	CGGGCUGUCCGGAGGGGUCGGCU	3.89	5.23	5.13	-1.34	-0.10
hsa-miR-3934	MIMAT0018349	UCAGGUGUGGAAACUGAGGCAG	2.39	3.73	3.77	-1.35	0.04
hsa-miR-557	MIMAT0003221	GUUUGCACGGGUGGGCCUUGUCU	2.39	3.73	3.77	-1.35	0.04

hsa-miR-515-5p	MIMAT0002826	UUCUCCAAAAGAAAGCACUUUCUG	2.39	3.73	3.42	-1.35	-0.31
hsa-miR-892a	MIMAT0004907	CACUGUGUCCUUUCUGCGUAG	2.39	3.73	3.42	-1.35	-0.31
hsa-miR-4448	MIMAT0018967	GGCUCCUUGGUCUAGGGGUA	2.61	3.98	3.66	-1.37	-0.31
hsa-miR-2682-5p	MIMAT0013517	CAGGCAGUGACUGUUCAGACGUC	3.61	4.98	4.66	-1.37	-0.31
hsa-miR-647	MIMAT0003317	GUGGCUGCACUCACUUCCUUC	2.80	4.18	3.96	-1.38	-0.22
hsa-miR-371a-3p	MIMAT0000723	AAGUGCCGCCAUCUUUUGAGUGU	2.97	4.36	4.05	-1.39	-0.31
hsa-miR-761	MIMAT0010364	GCAGCAGGGUGAAACUGACACA	3.61	5.03	4.82	-1.42	-0.21
hsa-miR-548an	MIMAT0019079	AAAAGGCAUUGUGGUUUUUG	2.39	3.86	3.77	-1.47	-0.09
hsa-miR-1273c	MIMAT0015017	GGCGACAAAACGAGACCCUGUC	2.39	3.86	3.42	-1.47	-0.44
hsa-miR-744-5p	MIMAT0004945	UGCGGGGCUAGGGCUAACAGCA	2.80	4.28	4.13	-1.47	-0.14
hsa-miR-487a	MIMAT0002178	AAUCAUACAGGGACAUCCAGUU	2.80	4.28	4.05	-1.47	-0.22
hsa-miR-584-5p	MIMAT0003249	UUAUGGUUUGCCUGGGACUGAG	2.97	4.44	4.42	-1.47	-0.02
hsa-miR-1278	MIMAT0005936	UAGUACUGUGCAUAUCAUCUAU	3.26	4.73	4.29	-1.47	-0.45
hsa-miR-626	MIMAT0003295	AGCUGUCUGAAAAUGUCUU	3.50	4.98	4.92	-1.47	-0.06
hsa-miR-217	MIMAT0000274	UACUGCAUCAGGAACUGAUUGGA	1.80	3.28	3.13	-1.48	-0.14
hsa-miR-497-5p	MIMAT0002820	CAGCAGCACACUGUGGUUUGU	2.12	3.60	3.29	-1.48	-0.31
hsa-miR-548ag	MIMAT0018969	AAAGGUAAUUGUGGUUUCUGC	3.26	4.80	4.49	-1.54	-0.31
hsa-miR-182-5p	MIMAT0000259	UUUGGCAAUGGUAGAACUCACACU	6.79	8.35	8.05	-1.56	-0.31
hsa-miR-640	MIMAT0003310	AUGAUCCAGGAACCUGCCUCU	2.61	4.18	4.29	-1.57	0.10
hsa-miR-520a-5p	MIMAT0002833	CUCCAGAGGGAAGUACUUUCU	2.61	4.18	3.87	-1.57	-0.31
hsa-miR-3154	MIMAT0015028	CAGAAGGGGAGUUGGGAGCAGA	2.12	3.73	3.42	-1.61	-0.31
hsa-miR-1468	MIMAT0006789	CUCCGUUUGCCUGUUUCGCUG	2.12	3.73	3.29	-1.61	-0.45
hsa-miR-504	MIMAT0002875	AGACCCUGGUCUGCACUCUAUC	2.80	4.44	4.36	-1.64	-0.09
hsa-miR-1245b-3p	MIMAT0019951	UCAGAUGAUCUAAAGGCCUAUA	1.80	3.44	3.55	-1.65	0.10
hsa-miR-499b-3p	MIMAT0019898	AACAUCACUGCAAGUCUUAACA	1.80	3.44	3.55	-1.65	0.10

hsa-miR-518f-3p	MIMAT0002842	GAAAGCGCUUCUCUUUAGAGG	1.80	3.44	3.13	-1.65	-0.31
hsa-miR-611	MIMAT0003279	GCGAGGACCCCUCGGGGUCUGAC	2.39	4.08	3.55	-1.70	-0.53
hsa-miR-767-3p	MIMAT0003883	UCUGCUCAUACCCCAUGGUUUCU	1.38	3.08	2.96	-1.70	-0.12
hsa-miR-369-5p	MIMAT0001621	AGAUCGACCGUGUUAUAUUCGC	1.38	3.08	2.77	-1.70	-0.31
hsa-miR-1268a	MIMAT0005922	CGGGCGUGGUGGUGGGGG	3.61	5.32	4.92	-1.71	-0.40
hsa-miR-922	MIMAT0004972	GCAGCAGAGAAUAGGACUACGUC	3.26	4.98	4.49	-1.72	-0.49
hsa-miR-329	MIMAT0001629	AACACACCUGGUUAACCUCUUU	2.80	4.52	4.21	-1.72	-0.31
hsa-miR-625-5p	MIMAT0003294	AGGGGGAAAGUUCUAUAGUCC	3.12	4.86	4.29	-1.74	-0.57
hsa-miR-216a	MIMAT0000273	UAAUCUCAGCUGGCAACUGUGA	2.61	4.36	4.05	-1.75	-0.31
hsa-miR-2053	MIMAT0009978	GUGUUAAUUAAACCUCUAUUUAC	2.61	4.36	3.87	-1.75	-0.49
hsa-miR-588	MIMAT0003255	UUGGCCACAAUGGGUUAGAAC	2.61	4.36	3.66	-1.75	-0.70
hsa-miR-1204	MIMAT0005868	UCGUGGCCUGGUCUCCAUUAU	2.39	4.18	3.77	-1.80	-0.41
hsa-miR-518d-3p	MIMAT0002864	CAAAGCGCUUCCCUUUGGAGC	2.39	4.18	3.42	-1.80	-0.76
hsa-miR-124-3p	MIMAT0000422	UAAGGCACGCGGUGAAUGCC	1.80	3.60	3.13	-1.80	-0.46
hsa-miR-509-3-5p	MIMAT0004975	UACUGCAGACGUGGCAAUCAUG	0.80	2.60	2.29	-1.80	-0.31
hsa-miR-516a-3p	MIMAT0006778	UGCUUCCUUUCAGAGGGU	2.97	4.80	4.13	-1.83	-0.66
hsa-miR-491-3p	MIMAT0004765	CUUAUGCAAGAUUCCCUUCUAC	2.12	3.98	3.29	-1.85	-0.69
hsa-miR-450b-5p	MIMAT0004909	UUUUGCAAUAUGUUCCUGAAUA	2.80	4.67	4.49	-1.87	-0.18
hsa-miR-1287	MIMAT0005878	UGCUGGAUCAGUGGUUCGAGUC	2.80	4.67	4.42	-1.87	-0.24
hsa-miR-548m	MIMAT0005917	CAAAGGUAUUUGUGGUUUUUG	2.39	4.28	3.66	-1.89	-0.61
hsa-miR-499b-5p	MIMAT0019897	ACAGACUUGCUGUGAUGUUCA	2.39	4.28	3.55	-1.89	-0.73
hsa-miR-518e-3p	MIMAT0002861	AAAGCGCUUCCCUUCAGAGUG	1.38	3.28	2.96	-1.89	-0.31
hsa-miR-203	MIMAT0000264	GUGAAAUGUUUAGGACCACUAG	2.61	4.52	3.66	-1.91	-0.86
hsa-miR-630	MIMAT0003299	AGUAUUCUGUACCAGGGAAGGU	12.47	14.39	13.81	-1.92	-0.58
hsa-miR-1227	MIMAT0005580	CGUGCCACCCUUUUCCCCAG	1.80	3.73	3.13	-1.94	-0.60

hsa-miR-1236	MIMAT0005591	CCUCUUCCCCUUGUCUCUCCAG	2.12	4.08	3.55	-1.96	-0.53
hsa-miR-1265	MIMAT0005918	CAGGAUGUGGUCAAGUGUUGUU	2.39	4.36	3.87	-1.98	-0.49
hsa-miR-585	MIMAT0003250	UGGGCGUAUCUGUAUGCUA	2.39	4.36	3.77	-1.98	-0.59
hsa-miR-559	MIMAT0003223	UAAAGUAAAUAUGCACCAAAA	2.12	4.18	3.55	-2.06	-0.63
hsa-miR-1225-3p	MIMAT0005573	UGAGCCCCUGUGCCGCCCCCAG	1.80	3.86	3.13	-2.06	-0.73
hsa-miR-2115-5p	MIMAT0011158	AGCUUCCAUGACUCCUGAUGGA	1.80	3.86	2.96	-2.06	-0.90
hsa-miR-548am-3p	MIMAT0019076	CAAAAACUGCAGUUACUUUUGU	1.80	3.98	3.87	-2.18	-0.10
hsa-miR-323a-5p	MIMAT0004696	AGGUGGUCCGUGGCGCGUUCGC	1.80	3.98	3.42	-2.18	-0.55
hsa-miR-654-3p	MIMAT0004814	UAUGUCUGCUGACCAUCACCUU	1.80	3.98	3.42	-2.18	-0.55
hsa-miR-302e	MIMAT0005931	UAAGUGCUUCCAUGCUU	2.12	4.36	3.66	-2.24	-0.70
hsa-miR-3175	MIMAT0015052	CGGGGAGAGAACGCAGUGACGU	0.80	3.08	3.42	-2.28	0.34
hsa-miR-455-5p	MIMAT0003150	UAUGUGCCUUUGGACUACAUCG	0.80	3.08	2.77	-2.28	-0.31
hsa-miR-193a-5p	MIMAT0004614	UGGGUCUUUGCGGGCGAGAUGA	2.61	4.92	4.29	-2.31	-0.63
hsa-miR-450b-3p	MIMAT0004910	UUGGGAUCAUUUUGCAUCCAUA	1.38	3.73	3.42	-2.35	-0.31
hsa-miR-1226-3p	MIMAT0005577	UCACCAGCCCUGUGUUCCCUAG	2.61	5.08	4.36	-2.47	-0.73
hsa-miR-1202	MIMAT0005865	GUGCCAGCUGCAGUGGGGGAG	2.12	4.60	3.77	-2.48	-0.83
hsa-miR-3136-5p	MIMAT0015003	CUGACUGAAUAGGUAGGGUCAUU	0.80	3.28	3.29	-2.48	0.01
hsa-miR-4485	MIMAT0019019	UAACGGCCGCGGUACCCUAA	-0.20	2.28	2.96	-2.48	0.69
hsa-miR-136-5p	MIMAT0000448	ACUCCAUUUGUUUUGAUGAUGGA	0.80	3.44	2.77	-2.65	-0.67
hsa-miR-502-3p	MIMAT0004775	AAUGCACCUGGGCAAGGAUUCA	0.80	3.44	2.77	-2.65	-0.67
hsa-miR-614	MIMAT0003282	GAACGCCUGUUCUUGCCAGGUGG	0.80	3.73	4.05	-2.94	0.32
hsa-miR-338-5p	MIMAT0004701	AACAAUAUCCUGGUGCUGAGUG	-0.20	2.86	2.77	-3.06	-0.09
hsa-miR-606	MIMAT0003274	AAACUACUGAAAAUCAAAGAU	-0.20	3.08	3.13	-3.28	0.05
hsa-miR-551a	MIMAT0003214	GCGACCCACUCUUGGUUUCCA	0.80	4.36	3.96	-3.56	-0.40

Footnotes:

* Values represent log_2 fold change of significantly up- or down-regulated miRNAs in EV71-infected mild symptom (n=4) and severe symptom (n=4) subjects from healthy control (n=4).

1. The blood serums were obtained from healthy children.
2. The blood serums were obtained from EV71-infected children with mild symptom.
3. The blood serums were obtained from EV71-infected children with severe symptom.
4. The up-regulated is defined as the expression level of miRNA is more or equal 2 folds detected in EV71-infected mild symptom in comparison with healthy control group.
5. The down-regulated is defined as the expression level of miRNA is less 2 folds detected in EV71-infected mild symptom in comparison with healthy control group

Table S3. The list of up- and down-regulated expression of miRNAs detected from the blood serum of EV71-infected patient with severe symptom in comparison with healthy controls.

Gene Name	Accession Number	Target Sequence (5' to 3')	Mild[2],*	Control[1],*	Severe[3],*	Severe vs. Control*	Mild vs. Control*
**<u>Up-Regulated</u>**[4]							
hsa-miR-876-5p	MIMAT0000269	UAACAGUCUCCAGUCACGGCC	4.50	4.36	7.61	3.25	0.14
hsa-miR-30c-5p	MIMAT0000244	UGUAAACAUCCUACACUCUCAGC	8.20	7.34	9.09	1.75	0.86
hsa-miR-580	MIMAT0003245	UUGAGAAUGAUGAAUCAUUAGG	3.50	2.60	4.21	1.61	0.90
hsa-miR-454-3p	MIMAT0003885	UAGUGCAAUAUUGCUUAUAGGGU	8.72	7.79	9.40	1.61	0.93
hsa-miR-296-5p	MIMAT0000690	AGGGCCCCCCCUCAAUCCUGU	6.90	6.52	8.11	1.59	0.38
hsa-miR-1271-5p	MIMAT0005796	CUUGGCACCUAGCAAGCACUCA	2.12	2.60	4.13	1.54	-0.48
hsa-let-7b-5p	MIMAT0000063	UGAGGUAGUAGGUUGUGUGGUU	13.07	12.75	14.26	1.51	0.31
hsa-miR-15b-5p	MIMAT0000417	UAGCAGCACAUCAUGGUUUACA	14.17	13.60	15.05	1.45	0.57
hsa-miR-339-5p	MIMAT0000764	UCCCUGUCCUCCAGGAGCUCACG	4.19	3.44	4.87	1.43	0.75
hsa-miR-369-3p	MIMAT0000721	AAUAAUACAUGGUUGAUCUUU	2.80	1.86	3.29	1.43	0.94
hsa-miR-513c-5p	MIMAT0005789	UUCUCAAGGAGGUGUCGUUUAU	2.61	1.86	3.29	1.43	0.75
hsa-let-7c	MIMAT0000064	UGAGGUAGUAGGUUGUAUGGUU	9.88	9.72	11.11	1.39	0.16
hsa-miR-331-3p	MIMAT0000760	GCCCCUGGGCCUAUCCUAGAA	5.78	5.13	6.52	1.39	0.64
hsa-miR-652-3p	MIMAT0003322	AAUGGCGCCACUAGGGUUGUG	8.77	8.24	9.52	1.28	0.53
hsa-miR-92a-3p	MIMAT0000092	UAUUGCACUUGUCCCGGCCUGU	11.30	10.83	12.10	1.28	0.47
hsa-miR-1539	MIMAT0007401	UCCUGCGCGUCCCAGAUGCCC	2.80	2.28	3.55	1.27	0.53
hsa-miR-4508	MIMAT0019045	GCGGGGCUGGGCGCGCG	7.33	6.38	7.61	1.22	0.95
hsa-let-7f-5p	MIMAT0000067	UGAGGUAGUAGAUUGUAUAGUU	9.57	8.86	10.07	1.21	0.71
hsa-miR-3195	MIMAT0015079	CGCGCCGGGCCCGGGUU	7.31	6.99	8.18	1.19	0.32
hsa-miR-552	MIMAT0003215	AACAGGUGACUGGUUAGACAA	3.26	2.60	3.77	1.17	0.66

hsa-miR-424-5p	MIMAT0001341	CAGCAGCAAUUCAUGUUUUGAA	3.39	3.44	4.61	1.16	-0.06
hsa-let-7a-5p	MIMAT0000062	UGAGGUAGUAGGUUGUAUAGUU	12.52	12.40	13.56	1.15	0.12
hsa-miR-26a-5p	MIMAT0000082	UUCAAGUAAUCCAGGAUAGGCU	11.07	10.13	11.28	1.15	0.95
hsa-miR-660-5p	MIMAT0003338	UACCCAUUGCAUAUCGGAGUUG	9.57	8.73	9.87	1.14	0.83
hsa-miR-3144-5p	MIMAT0015014	AGGGGACCAAAGAGAUAUAUAG	3.12	2.86	3.96	1.10	0.26
hsa-let-7e-5p	MIMAT0000066	UGAGGUAGGAGGUUGUAUAGUU	7.39	6.68	7.76	1.08	0.71
hsa-miR-107	MIMAT0000104	AGCAGCAUUGUACAGGGCUAUCA	9.99	9.25	10.26	1.01	0.74
hsa-miR-3196	MIMAT0015080	CGGGGCGGCAGGGGCCUC	3.12	2.86	3.87	1.01	0.26
hsa-miR-550a-5p	MIMAT0004800	AGUGCCUGAGGGAGUAAGAGCCC	2.12	2.86	3.87	1.01	-0.74
hsa-miR-490-3p	MIMAT0002806	CAACCUGGAGGACUCCAUGCUG	2.39	2.28	3.29	1.01	0.11
**Down-Regulated**[5]							
hsa-miR-629-5p	MIMAT0004810	UGGGUUUACGUUGGGAGAACU	6.47	7.36	6.34	-1.02	-0.89
hsa-miR-876-5p	MIMAT0004924	UGGAUUUCUUUGUGAAUCACCA	3.12	2.60	1.55	-1.05	0.53
hsa-miR-9-5p	MIMAT0000441	UCUUUGGUUAUCUAGCUGUAUGA	3.61	4.18	3.13	-1.05	-0.57
hsa-miR-502-5p	MIMAT0002873	AUCCUUGCUAUCUGGGUGCUA	3.12	3.60	2.55	-1.05	-0.47
hsa-miR-889	MIMAT0004921	UUAAUAUCGGACAACCAUUGU	2.80	3.60	2.55	-1.05	-0.80
hsa-miR-920	MIMAT0004970	GGGGAGCUGUGGAAGCAGUA	2.97	3.86	2.77	-1.09	-0.89
hsa-miR-517a-3p	MIMAT0002852	AUCGUGCAUCCCUUUAGAGUGU	3.71	4.52	3.42	-1.10	-0.82
hsa-miR-342-3p	MIMAT0000753	UCUCACACAGAAAUCGCACCCGU	9.57	9.26	8.11	-1.14	0.31
hsa-miR-873-5p	MIMAT0004953	GCAGGAACUUGUGAGUCUCCU	3.39	3.44	2.29	-1.16	-0.06
hsa-miR-146b-3p	MIMAT0004766	UGCCCUGUGGACUCAGUUCUGG	3.80	4.44	3.29	-1.16	-0.64
hsa-miR-1262	MIMAT0005914	AUGGGUGAAUUUGUAGAAGGAU	4.05	4.60	3.42	-1.17	-0.55
hsa-miR-936	MIMAT0004979	ACAGUAGAGGGAGGAAUCGCAG	3.50	4.36	3.13	-1.23	-0.86
hsa-miR-3182	MIMAT0015062	GCUUCUGUAGUGUAGUC	3.50	4.44	3.13	-1.31	-0.94

hsa-miR-199a-3p+hsa-miR-199b-3p	MIMAT0000232	ACAGUAGUCUGCACAUUGGUUA	6.81	6.40	5.09	-1.31	0.41
hsa-miR-1181	MIMAT0005826	CCGUCGCCGCCACCCGAGCCG	4.44	4.86	3.55	-1.31	-0.42
hsa-miR-3127-5p	MIMAT0014990	AUCAGGGCUUGUGGAAUGGGAAG	2.97	3.86	2.55	-1.31	-0.89
hsa-miR-769-3p	MIMAT0003887	CUGGGAUCUCCGGGGUCUUGGUU	3.97	4.36	2.96	-1.40	-0.39
hsa-miR-219-2-3p	MIMAT0004675	AGAAUUGUGGCUGGACAUCUGU	3.71	4.36	2.96	-1.40	-0.66
hsa-miR-125a-5p	MIMAT0000443	UCCCUGAGACCCUUUAACCUGUGA	4.56	4.18	2.77	-1.41	0.37
hsa-miR-2054	MIMAT0009979	CUGUAAUAUAAAUUUAAUUUAUU	2.80	3.44	1.96	-1.48	-0.64
hsa-miR-361-5p	MIMAT0000703	UUAUCAGAAUCUCCAGGGGUAC	6.46	7.42	5.85	-1.58	-0.97
hsa-miR-337-5p	MIMAT0004695	GAACGGCUUCAUACAGGAGUU	3.89	4.73	3.13	-1.60	-0.85
hsa-miR-675-5p	MIMAT0004284	UGGUGCGGAGAGGGCCCACAGUG	2.39	3.28	1.55	-1.72	-0.89
hsa-miR-346	MIMAT0000773	UGUCUGCCCGCAUGCCUGCCUCU	3.71	4.08	2.29	-1.80	-0.38
hsa-miR-758	MIMAT0003879	UUUGUGACCUGGUCCACUAACC	3.12	3.44	1.55	-1.89	-0.32
hsa-miR-27a-3p	MIMAT0000084	UUCACAGUGGCUAAGUUCCGC	4.89	5.03	3.13	-1.90	-0.14
hsa-miR-221-3p	MIMAT0000278	AGCUACAUUGUCUGCUGGGUUUC	7.37	6.70	4.77	-1.93	0.67
hsa-miR-1973	MIMAT0009448	ACCGUGCAAAGGUAGCAUA	5.84	6.65	4.55	-2.10	-0.81
hsa-miR-1910	MIMAT0007884	CCAGUCCUGUGCCUGCCGCCU	2.97	3.08	0.96	-2.12	-0.11
hsa-miR-150-5p	MIMAT0000451	UCUCCCAACCCUUGUACCAGUG	10.85	10.97	6.64	-4.34	-0.12

Footnotes:

* Values represent fold change (log_2) of significantly up- or down-regulated miRNAs in EV71-infected mild symptom (n=4) and severe symptom (n=4) subjects from healthy control (n=4).

1. The blood serums were obtained from healthy children.
2. The blood serums were obtained from EV71-infected children with mild symptom.
3. The blood serums were obtained from EV71-infected children with severe symptom.

4. The up-regulated is defined as the expression level of miRNA is more or equal 2 folds detected in EV71-infected severe symptom in comparison with healthy control group.
5. The down-regulated is defined as the expression level of miRNA is less 2 folds detected in EV71-infected severe symptom in comparison with healthy control group

Fig. S1. MicroRNA-mediated regulation of target genes in response to EV71 infection mild symptom. KEGG pathway analysis was performed on cellular genes identified as important for EV71 progressive disease. The red stars were located near the targeted genes of miR-494.

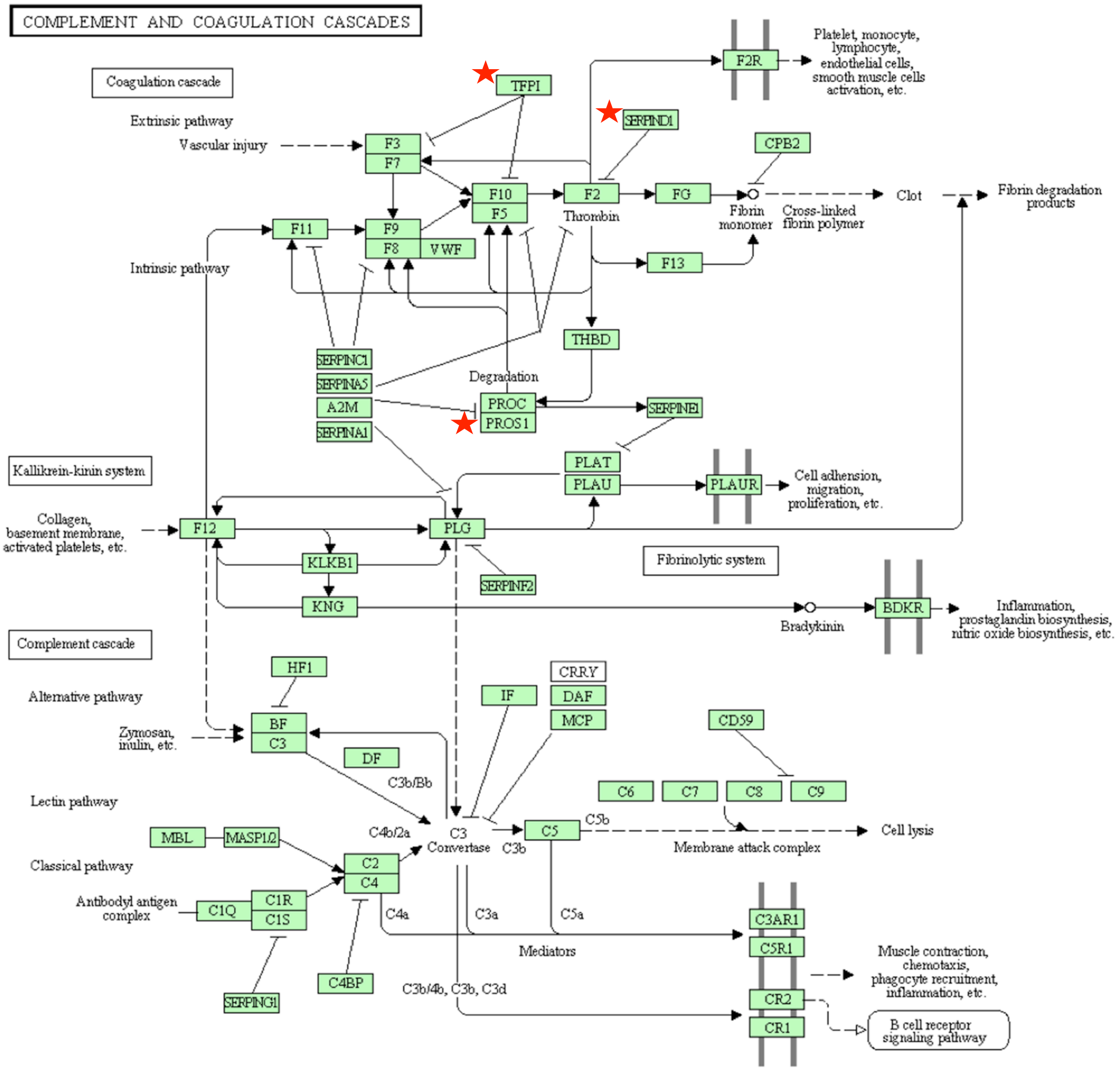

Fig. S2. MicroRNA-mediated regulation of target genes in response to EV71 infection mild symptom. KEGG pathway analysis was performed on cellular genes identified as important for EV71 progressive disease. The red stars were located near the targeted genes of miR-551a.

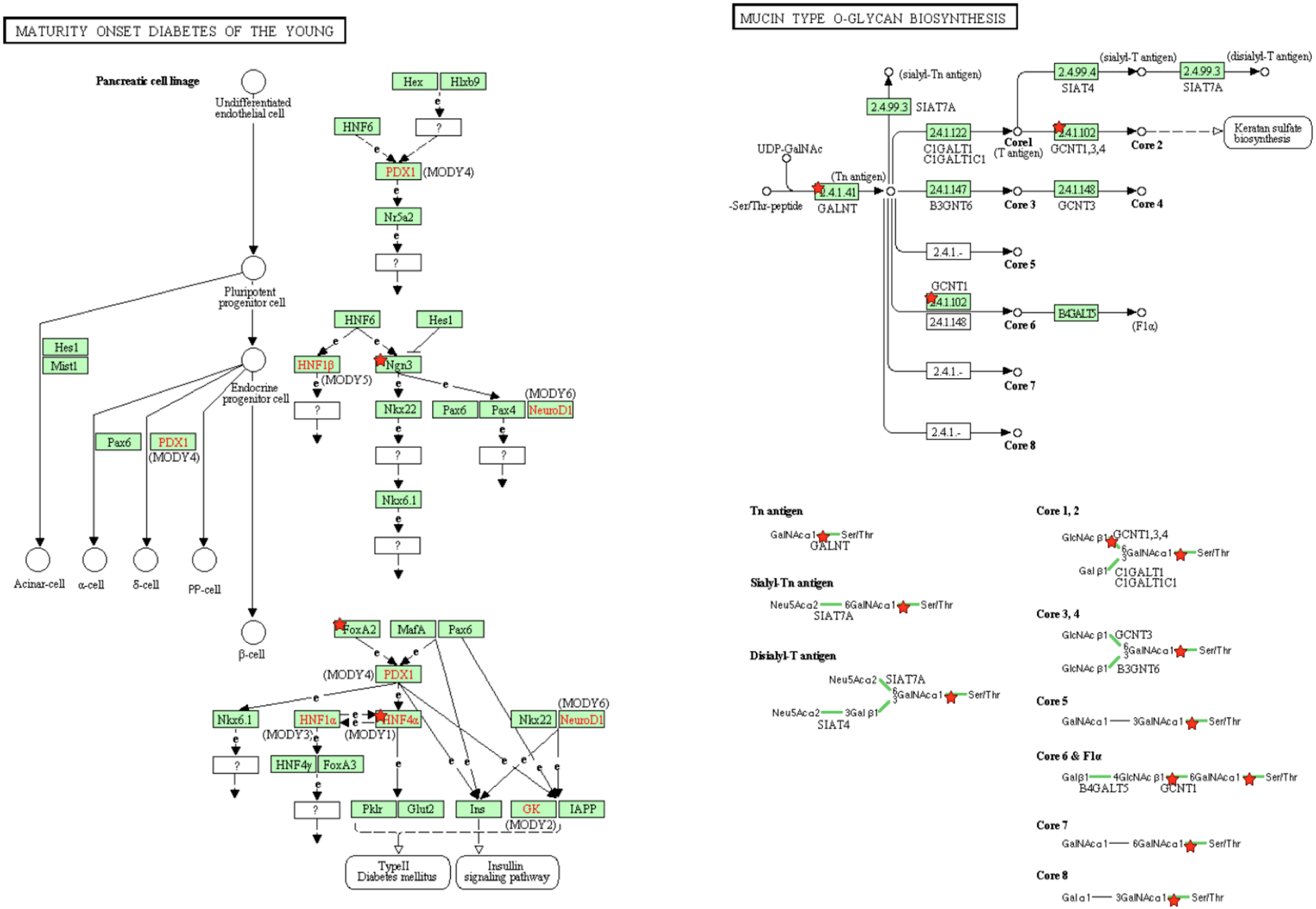

Fig. S3. MicroRNA-mediated regulation of target genes in response to EV71 infection mild symptom. KEGG pathway analysis was performed on cellular genes identified as important for EV71 progressive disease. The red stars were located near the targeted genes of miR-876-5p.

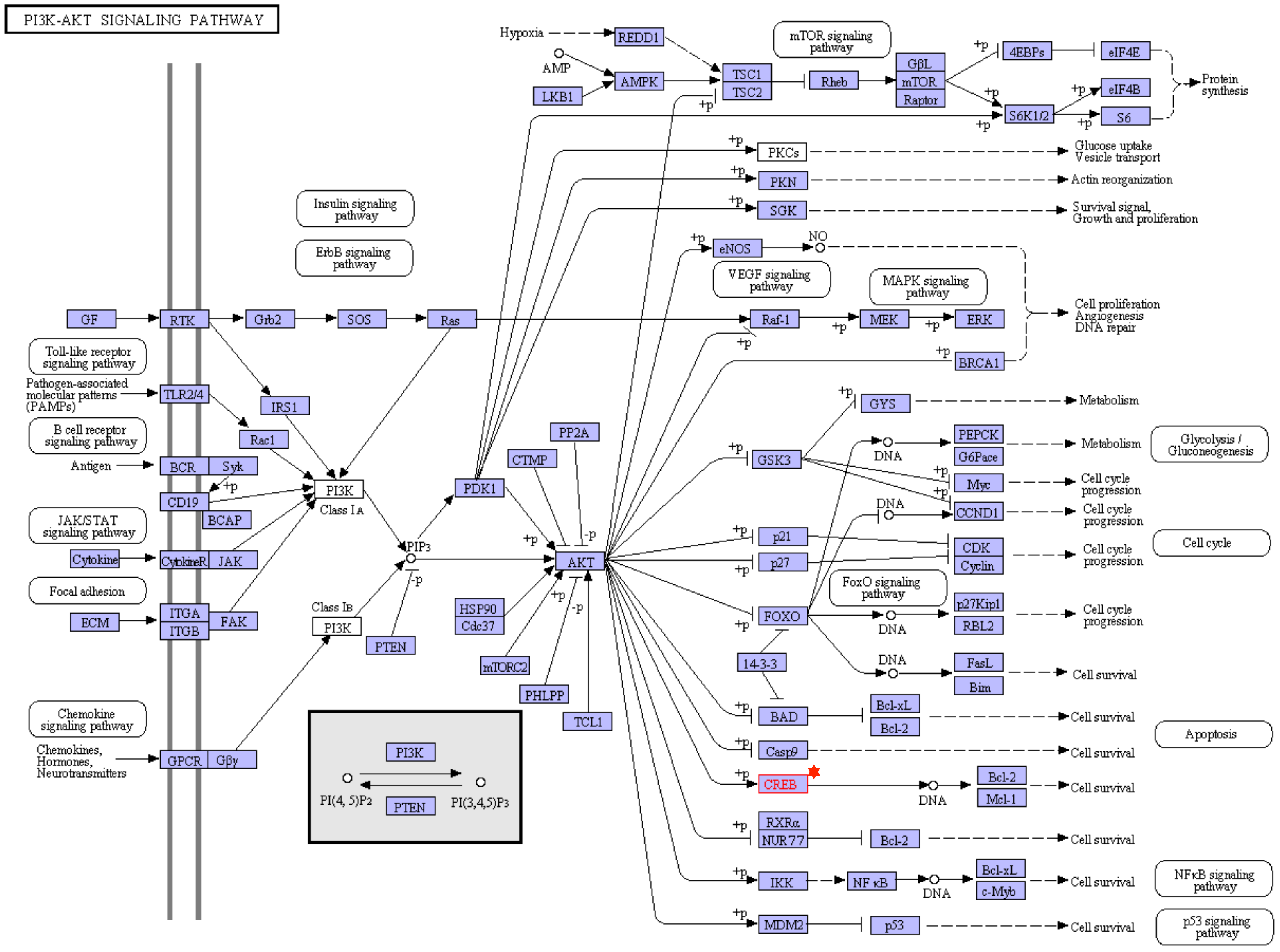

Fig. S4. MicroRNA-mediated regulation of target genes in response to EV71 infection mild symptom. KEGG pathway analysis was performed on cellular genes identified as important for EV71 progressive disease. The red stars were located near the targeted genes of miR-150-5p.

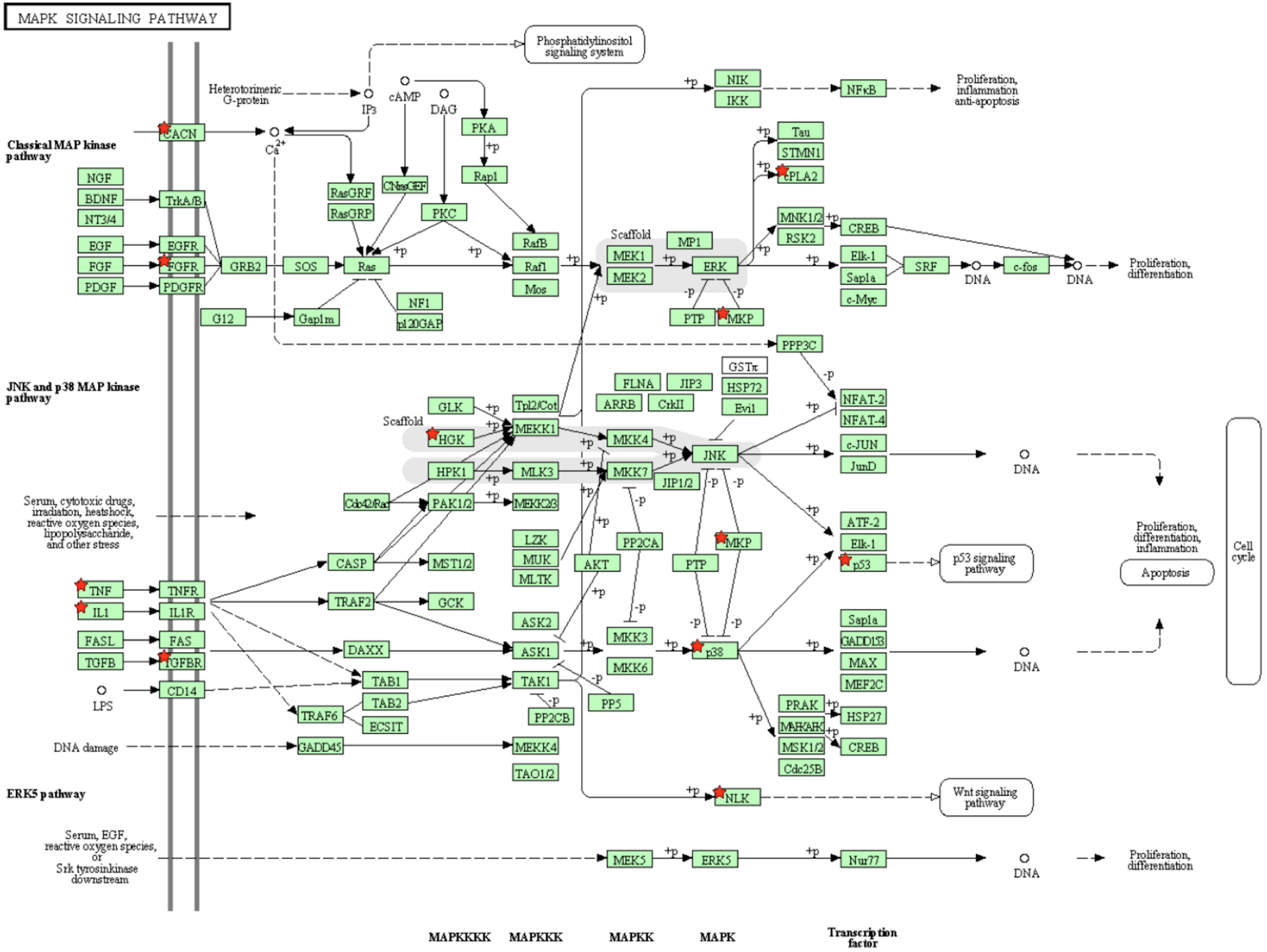

Supplement: Supplementary Information [file srep24149-s1.pdf]
